# 1294. A Scientometric Study of the Top 100 Most Influential Papers on Septic Arthritis of Native Joints

**DOI:** 10.1093/ofid/ofad500.1133

**Published:** 2023-11-27

**Authors:** Berna Karaismailoglu, Ali Egemen Koroglu, Arin Celayir, Bedri Karaismailoglu

**Affiliations:** Basaksehir Cam and Sakura City Hospital, Boston University, Istanbul, Istanbul, Turkey; Istanbul University-Cerrahpasa, Istanbul, Istanbul, Turkey; Istanbul University-Cerrahpasa, Istanbul, Istanbul, Turkey; Istanbul University-Cerrahpasa, Istanbul, Istanbul, Turkey

## Abstract

**Background:**

The rising popularity of bibliometric studies stems from their ability to determine the features of articles on particular topics. The objective of this study was to identify and evaluate the characteristics of the top 100 highly cited papers on septic arthritis of the native joints.

**Methods:**

The study involved an analysis of the Web of Science database to identify the 100 most-cited articles related to septic arthritis. The characteristics of these articles, such as publication year, country of origin, journal, study type, and funding availability, were recorded and analyzed to identify any relationships or correlations with total citation count or citation density (i.e., citations per year). Additionally, software was used to create a visualization of the most frequently used keywords in the articles.

**Results:**

The article with the highest citation count and density had 309 and 21.6, respectively. Out of the 100 selected papers, the majority (44 articles) were contributed by the United States. Case series studies were the most common type of research with 26 articles. Annals of the Rheumatic Diseases published the highest number of articles (8 in total). 15 studies were funded. The analysis revealed that the citation density of review articles was significantly higher than that of clinical and basic science studies (p< 0.001). The citation density was positively correlated with the publication year and number of contributing institutions, but negatively correlated with the level of evidence.

**Figure 1**

**Figure:**
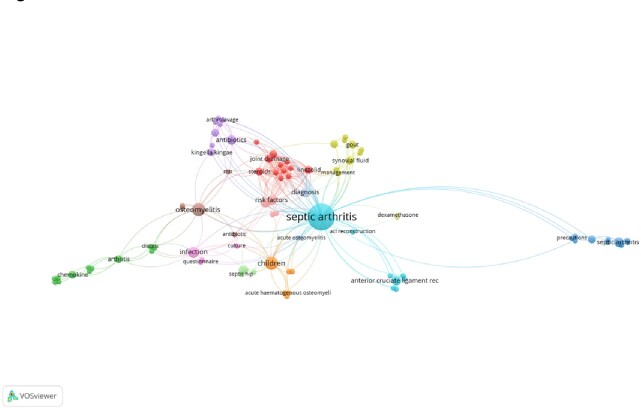

The map of keyword co-occurrence obtained by VOSviewer Version 1.6.16.

**Figure 2**

**Figure:**
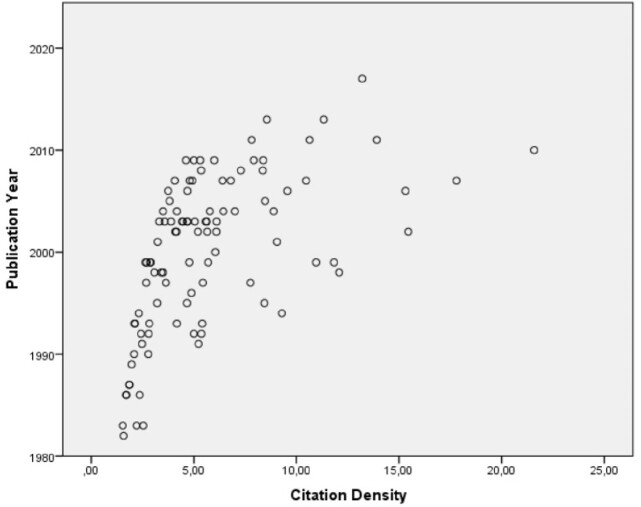

The correlation analysis between citation density and the publication year. A positive correlation was found indicating increased citation density in more recent papers. (Spearman’s Rho =0.655, p<0.001)

**Figure 3**

**Figure:**
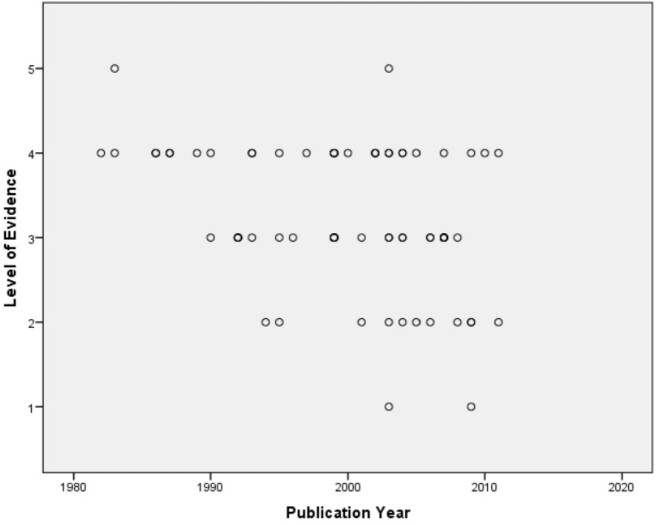

The correlation analysis between level of evidence and publication year. A negative correlation was found indicating that the recent papers have higher level of evidence. (Spearman’s Rho=-350, p=0.003)

**Conclusion:**

The study found that the majority of highly cited papers related to septic arthritis have a low level of evidence, highlighting the need for higher quality research in this area. Furthermore, the analysis revealed a positive correlation between citation density and both publication year and number of institutions involved, indicating better citation performance for more recent articles and those with contributions from multiple institutions. There was a negative correlation between level of evidence and publication year, suggesting that study designs have improved over time.

**Disclosures:**

**All Authors**: No reported disclosures

